# TiRobot‑assisted versus conventional fluoroscopy-assisted percutaneous sacroiliac screw fixation for pelvic ring injuries: a meta‑analysis

**DOI:** 10.1186/s13018-022-03420-x

**Published:** 2022-12-05

**Authors:** Chunpeng Zhao, Gang Zhu, Yu Wang, Xinbao Wu

**Affiliations:** 1 Department of Orthopedics and Traumatology, Beijing Jishuitan Hospital, Beijing, 100035 China; 2 Rossum Robot Co., Ltd., Beijing, 100083 China; 3 School of Biological Science and Medical Engineering, Beihang University, Beijing, 100083 China; 4 Beijing Advanced Innovation Center for Biomedical Engineering, Beihang University, Beijing, 100083 China

**Keywords:** Pelvic fracture, TiRobot, Robot-assisted, Percutaneous screw fixation

## Abstract

**Background:**

The TiRobot is the only robot that has been reported in the literature for posterior pelvic injuries. We aim to compare TiRobot-assisted pelvic screw fixation with the conventional fluoroscopy-assisted percutaneous sacroiliac screw fixation.

**Methods:**

We conducted a meta-analysis to identify studies involving TiRobot‑assisted versus conventional percutaneous sacroiliac screw fixation for pelvic ring injuries in electronic databases, including Web of Science, Embase, PubMed, Cochrane Controlled Trials Register, Cochrane Library, Highwire, CBM, CNKI, VIP, and WanFang database, up to April 2022. The following keywords were used: “TiRobot,” “robot,” “robotic,” “pelvic fracture,” “screw fixation,” “percutaneous,” and “pelvic ring injury.” Pooled effects of this meta-analysis were calculated using STATA SE version 15.0.

**Results:**

Compared with conventional fluoroscopy-assisted percutaneous sacroiliac screw fixation, TiRobot will result in less radiation exposure time of screw implantation (*P* = 0.000), less frequency of intraoperative fluoroscopy (*P* = 0.000), fewer guide wire attempts (*P* = 0.000), less intraoperative blood loss (*P* = 0.005), better screw accuracy (*P* = 0.011), better Majeed score (*P* = 0.031), and higher overall excellent and good rates of Majeed score (*P* = 0.018). However, there were no significant differences in terms of operative time (*P* = 0.055), fracture healing time (*P* = 0.365), and overall excellent and good rate of reduction accuracy (*P* = 0.426) between the two groups.

**Conclusion:**

TiRobot-assisted fixation has less intraoperative fluoroscopy and intraoperative blood loss, superior screw accuracy, and Majeed score compared with conventional percutaneous sacroiliac screw fixation. TiRobot has no significant effect on operative time, fracture healing time, and reduction accuracy. Given the relevant possible biases in our meta-analysis, we required more adequately powered and better-designed RCT studies with long-term follow-up to reach a firmer conclusion.

## Introduction

Pelvic fractures constitute up to 2–8% of all skeletal fractures. Pelvic fractures are usually unstable and are caused by high-energy trauma with a disability rate of 60% and a mortality rate of more than 13% [1, 2]. Unstable pelvic ring fractures often require simultaneous fixation of both the anterior and posterior pelvic rings to avoid serious complications, including chronic pain and sexual, bowel and bladder dysfunction [3–5].

Traditional fixation methods include open reduction internal fixation with plates from the anterior and posterior pathways, external fixation, and minimally invasive percutaneous screw fixation [5, 6]. With the advancement of medical technology and the popularization of minimally invasive concepts, the clinical application of open reduction and plate fixation is less frequent at present [6]. The percutaneous IS screw fixation is becoming increasingly popular worldwide with the advantages of less surgical trauma, less bleeding, fewer infection rates, fewer complications, earlier mobilization, and benefits for second-stage anatomical reconstruction surgery [6]. However, due to the unique anatomical structure of the posterior pelvic ring and the numerous influencing factors during C-arm fluoroscopies, such as morbidly obese patients or bowel gas, it is challenging to ensure the best position of each screw through manual operation under X‐ray monitoring [7]. Only a 4°deviation during the iliosacral screw insertion can lead to penetration of the anterior cortex of the sacrum or S1 foramina [8]. Several studies showed that the malposition rates of IS screw under traditional fluoroscopic guide were 2–15%, and the neurologic injury rates were 1–7% [8, 9]. Another limitation is that static two-dimensional (2D) intraoperative fluoroscopic imaging is inadequate for three-dimensional (3D) fragment alignments, necessitating repeat intraoperative images, which leads to prolonged radiation exposure to the patient and medical staff [10].

Furthermore, up to 44% of patients have Sacral 1 dysmorphism, which may influence the position of the screw [11, 12]. Percutaneous insertion is a highly demanding and challenging operative technique due to the complex pelvic anatomy, requiring skillful correlation of fluoroscopic images and bony landmarks [13]. Therefore, we need to find a safe and effective method to solve these problems.

Robotics and artificial intelligence applied to orthopedics have become an exciting topic. Various robot-assisted orthopedic surgery (RAOS) is currently available on the market. Each addresses specific surgeries and is characterized by special features that may involve different requirements or modus operandi. RAOS has been utilized in a variety of orthopedic operations, including fracture fixation for traumatic surgery, total hip and total knee arthroplasty [14, 15], spine surgery [16, 17], bone tumor surgery [18], arthroscopy [19], and fracture fixation for traumatic surgery [20]. However, few RAOS focus on screw implantation in pelvic fractures. The third RAOS, “TiRobot” (TINAVI Medical Technologies Co. Ltd., Beijing, China) from China has been certified by the China Food and Drug Administration (CFDA). It is the only robot that has been reported in the literature for posterior pelvic injuries. To our knowledge, there has been only one meta-analysis comparing TiRobot‑assisted versus fluoroscopy-assisted percutaneous sacroiliac screw fixation for pelvic ring injuries. However, the previous meta-analysis only included four observational studies, and all included studies are English articles. Considering that the TiRobot is only used in China, the restriction of the previous meta-analysis to English-language publications potentially limits the power that could be obtained with the inclusion of patient enrollment from Chinese-language studies. Finally, the previous meta-analysis did not do the pooled analysis of screw accuracy, reduction accuracy, and overall excellent and good rates of Majeed score. Thus, based on the current studies comparing TiRobot‑assisted versus fluoroscopy-assisted percutaneous sacroiliac screw fixation. We performed a meta-analysis, which includes English studies and Chinese studies. Moreover, our results included pooled analysis of screw accuracy, reduction accuracy, and overall excellent and good rates of Majeed score, which would provide a more exact conclusion and could supplement the previous meta-analysis.

## Methods

The current meta-analysis was registered on PROSPERO (International prospective register of systematic reviews), and the registration number was CRD42022318898. We strictly followed the PRISMA (preferred reporting items for systematic review and meta-analysis) guidelines to conduct this analysis according to the Preferred Reporting Items for Systematic Reviews and Meta-Analyses statement [21].

### Search strategy

We identified studies involving TiRobot‑assisted and fluoroscopy-assisted percutaneous sacroiliac screw fixation for pelvic ring injuries in electronic databases up to April 2022. The databases included Web of Science, Embase, PubMed, Cochrane, Controlled Trials Register, Cochrane Library, Highwire, CBM, CNKI, VIP, and WanFang database up to April 2022. The keywords used were “TiRobot,” “robot,” “robotic,” “pelvic fracture,” “screw fixation,” “percutaneous,” and “pelvic ring injury” in conjunction with Boolean operators “AND” or “OR.” There was no limitation on publication dates and language. We also reviewed the references of included studies and previous systematic reviews to identify other published or unpublished studies.

### Inclusion criteria

All randomized controlled trials (RCTs) and non-randomized controlled trials (non-RCTs) comparing TiRobot‑assisted and fluoroscopy-assisted percutaneous sacroiliac screw fixation for pelvic ring injuries were identified and included in the search strategy. These studies should meet the following inclusion criteria: (1) All papers with data on pelvic fracture were included. (2) TiRobot‑assisted percutaneous sacroiliac screw fixation for pelvic ring injuries was involved. (3) The comparator was conventional fluoroscopy-assisted percutaneous sacroiliac screw fixation in the original comparative study. (4) At least one of the following indexes was reported: radiation exposure time of screw implantation, frequency of intraoperative fluoroscopy, guide wire attempts, intraoperative blood loss, operative time, fracture healing time, screw accuracy, Majeed score, overall excellent and good rates of Majeed score and reduction accuracy. The operative time was defined from the start after sterile draping to the end of skin closure, which means that for the robot group, the time for robot preparation is also included in the intraoperative time. During each X-ray exposure, the fluoroscopy system calculated and displayed the real-time exposure seconds. The total fluoroscopy time of each screw implantation was recorded, which included the fluoroscopy for verifying the correct position of the screw placement after screw-setting. Intraoperative blood loss is the sum of the amount of blood through the suction apparatus and the bleeding volume at the gauze. We also excluded studies that had unclear or incomplete sample data.

### Data extraction process

Two independent researchers scanned the titles and abstracts of all literature searched, and they independently extracted the available data from each study. After excluding the trials which did not meet the inclusion criteria, we read the full text of the literature that might meet the inclusion criteria to determine whether this literature ultimately met the inclusion criteria. We extracted data based on the following: (1) research features (i.e., authors, year of publication, type of study, and patient population), (2) population information (i.e., number of patients, gender, age, BMI, and type of injury), (3) pelvic fracture information (i.e., fracture type, comorbidity, the average time from injury to surgery), and (4) outcome information. We addressed the disagreements by discussion or by involving a third author. We consulted the corresponding author to request missing data. If the necessary results are omitted, the first author will contact the authors by email or other means to obtain more data if necessary.

### Assessment of studies

Two authors independently evaluated the methodological quality. We assessed the non-randomized studies using the nine-star Newcastle–Ottawa Scale (NOS), a validated tool suitable for evaluating the quality of non-randomized studies [22]. According to the Cochrane Handbook for Systematic Reviews of Interventions [23], the methodological quality and basis of the RCTs were assessed as follows: randomization, allocation concealment, blind method, selective reporting, group similarity at baseline, incomplete outcome data, compliance, timing of outcome assessments, and intention-to-treat analysis. Two researchers independently assessed the studies, and disagreements between them were resolved through discussions with a third author or consensus.

### Ethical consideration

Ethical approval is not required because this study is based on the existing literature. The findings of this systematic review will be disseminated through a peer-reviewed journal.

### Statistical analysis

Heterogeneity was evaluated using the chi-square statistic and was quantified using *I*^2^. *P* values ≤ 0.1 or *I*^2^ value > 50% suggested high heterogeneity. Thus we used the randomized-effects model. Otherwise, we used the fixed-effects model [21]. In each study, we used the relative risk (RR) and relevant 95% confidence interval (CI) to measure dichotomous variables such as overall excellent and good rates of Majeed score, reduction accuracy, and screw accuracy. The mean difference (MD) or standard MD was used to assess continuous outcomes such as radiation exposure time of screw implantation, frequency of intraoperative fluoroscopy, guide wire attempts, intraoperative blood loss, operative time, fracture healing time, and Majeed score with a 95% confidence interval (CI). We used statistical algorithms to estimate the standard deviation for those studies that provided only continuous variables for means and range [24]. If *P* values were less than 0.05, we considered the results as a statistically significant difference. Sensitivity analysis was used to assess the stability of the results (if necessary). Pooled effects of this meta-analysis were calculated using STATA SE version 15.0 (The Nordic Cochrane Center, The Cochrane Collaboration, Copenhagen, Denmark).

## Results

### Search results

**Fig. 1:**
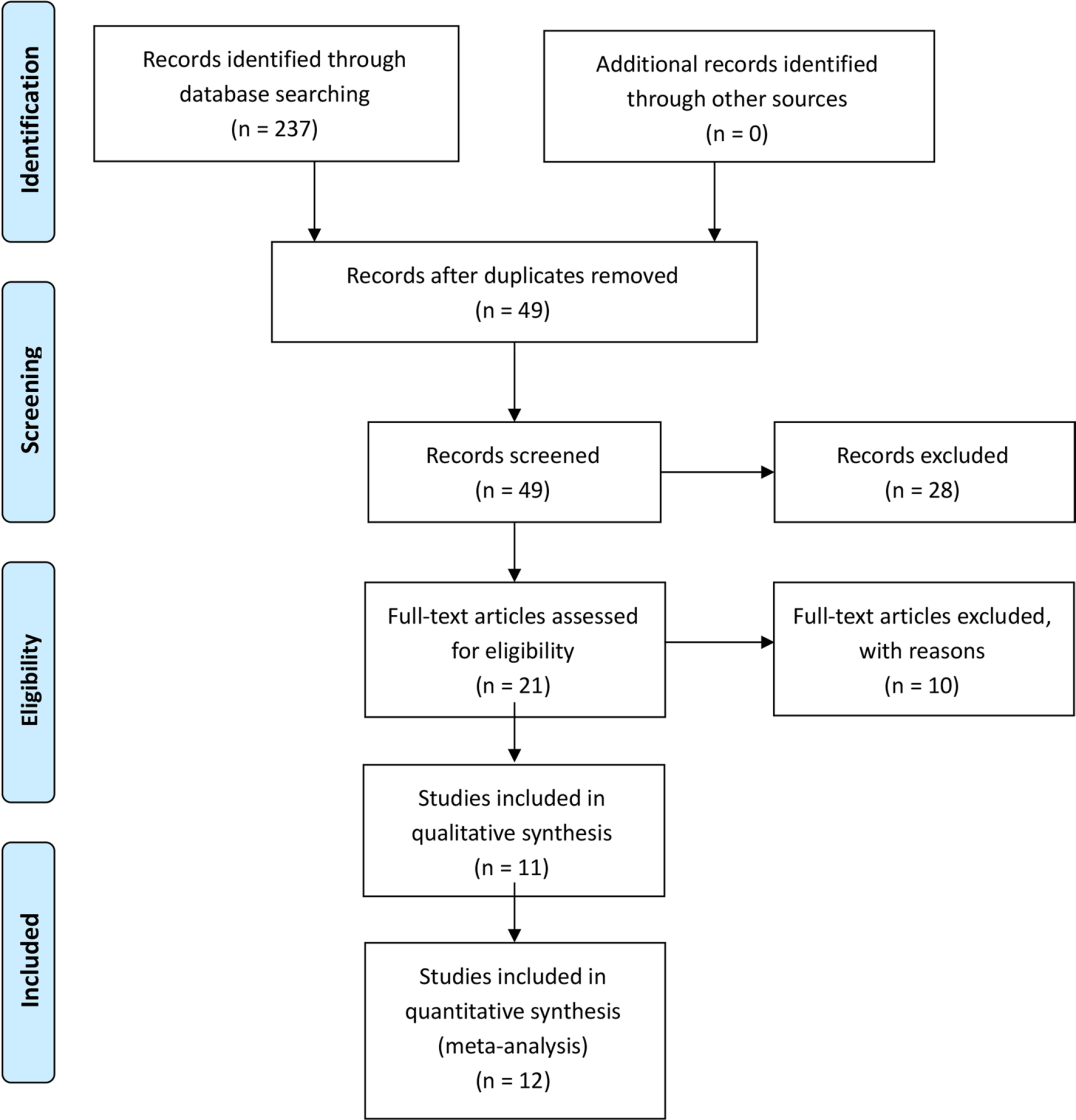
We identified 237 relevant citations from the databases. After deleting 188 duplicates, we obtained 49 articles. After reviewing the titles and abstracts of the 49 remaining articles, 28 irrelevant clinical studies were excluded. By reading the 21 full-text articles, we excluded another 10. The remaining 11 articles were deemed appropriate

The literature search and selection process are shown in Fig. 1. Finally, eleven publications from 2017 to 2022 were included in our meta-analysis. The detailed literature screening process is shown in the PRISMA flow diagram in Fig. 1. We identified 237 relevant citations from the databases according to the literature search strategy described earlier. After deleting 188 duplicates, we obtained 49 articles. After reviewing the titles and abstracts of the 49 remaining articles, 28 irrelevant clinical studies were excluded. By reading the 21 full-text articles, we excluded another ten for the following reasons: (systematic) reviews, non-compare groups, cadaver research, animal research, and no useful outcome data. The remaining eleven articles were deemed appropriate. Finally, we identified 628 patients assessed in 2 RCTs [25, 26] and 9 non-RCTs [27–35]

In Wang’s study [34], two subgroups compared TiRobot‑assisted versus conventional fluoroscopy-assisted percutaneous sacroiliac screw fixation. One is the S1 group (only S1 screws were implanted), and the other is the S2 group (only S2 screws were implanted) according to the location of the implanted screw. So we divided the study into two groups, Wang 2020(S1) and Wang 2020(S2).

### Study characteristics and quality

The detailed baseline characteristics and general intervention information are presented in Tables 1, 2, 3, and 4. The articles were published in English and Chinese between 2017 and April 2022.

**Table 1:** Summary of studies’ characteristics

Summary of studies’ characteristics			
References		Type of study	Patient population
Gu et al. [28]		RCS	Pelvic and acetabular fracture
Han et al. [32]		RCS	Posterior pelvic ring fracture
Hu et al. [27]		RCS	Posterior pelvic ring fracture
Li et al. [29]		RCS	Pelvic ring fractures
Liu et al. [33]		PCS	Pelvic ring fractures
Liu et al. [30]		RCS	Posterior pelvic ring fracture
Long et al. [31]		PCS	Posterior pelvic ring fracture
Wang et al. [25]		RCT	Posterior pelvic ring fracture
Wang et al. [34] (S1)		RCS	Posterior pelvic ring fracture
Wang et al. [34] (S2)		RCS	Posterior pelvic ring fracture
Wang et al. [35]		RCS	Pelvic ring fractures
Zhao et al. [26]		RCT	Posterior pelvic ring injuries

*RCT* randomized control trial; *RCS* retrospective cohort study; *PCS* prospective cohort study. Summary of studies’ characteristics including year of publication, study design, and patient population

**Table 2:** Summary of patient demographic details for each study

Author	TiRobot/conventional				
	Patients	Mean age (years)	Female gender (%)	BMI	Type of injury
Gu [28]	15/15	72/79	26.7/40	22.48/2.13	Fall (4/3), traffic accident (7/7), high‐level fall (3/3); crush (1/2)
Han et al. [32]	38/25	38.66/40.08	71.1/76	25.85/24.72	NA
Hu et al. [27]	12/12	42.3/43.9	33.3/41.7	28.73/28.19	Traffic accident (7/6), high‐level fall (3/3); crush (2/3)
Li et al. [29]	47/48	40.3/41.5	70.2/27.1	NA	Traffic accident (10/8), high‐level fall (21/23); crush (16/17)
Liu et al. [33]	24/21	37.4/39.8	37.5/40	29.8/28.9	Traffic accident (10/11), high‐level fall (8/7); crush (6/3)
Liu et al. [30]	10/32	36/34	20/25	NA	Total: Traffic accident (34), high‐level fall (6); Other (2)
Long et al. [31]	56/35	35.95/35.68	42.9/40	NA	Traffic accident (39/15), high‐level fall (14/19); Crush (3/1)
Wang et al. [25]	23/22	36/43	33/46.6	NA	NA
Wang et al. [34] (S1)	59/29	44.02/43.66	39.35/42.5	NA	NA
Wang et al. [34] (S2)	26/14	39.35/42.5	26.9/35.7	NA	NA
Wang et al. [35]	32/21	40.8/39.9	43.75/47.6	22.9/23.8	Traffic accident (12/8), high‐level fall (12/7); Crush (8/6)
Zhao et al. [26]	7/5	39/42	28.6/40	19.37/20.21	NA

Abbreviations: BMI, Body Mass Index. Summary of patient demographic information for each study including number of patients, gender, age, BMI, and type of injury

**Table 3:** Detailed characteristics of pelvic fracture information

Author	TiRobot/conventional or total		
	Fracture type	Comorbidity	Average time from injury to surgery(d)
Gu [28]	NA	NA	4.5/5.25
Han et al. [32]	Tile Type B (15/11), Type C (23/14)	NA	9.63/8.28
Hu et al. [27]	Denis Type I (6/8), Type II (6/4)	Hemorrhagic shock (4/1), spleen rupture (1/3), urethral injury (2/0), limb or rib fractures (5/4)	NA
Li et al. [29]	Tile Type B (23/21), Type C (24/27)	Limb fracture with hemorrhagic shock (21/22), bladder injury (1/3); pneumothorax or hemothorax (5/4); thoracolumbar vertebral fracture (11/8); craniocerebral injury (9/11)	NA
Liu et al. [33]	Tile Type B (17/14), Type C (7/7)	Rib fractures (9), thoracolumbar fractures (6), urethral ruptures (5)	5.2/5.7
Liu et al. [30]	Tile Type C (42)	Lumbar vertebrae fracture (8); femur fracture (5); Morel–Lavallée injury (3); tibial fracture (3); thoracic injury (2); cervical vertebral fracture (1); calcaneal fracture (2)	NA
Long et al. [31]	AO Type B (21/15), Type C (35/20)	Combined injury (40), shock (33)	9.61/9.71
Wang et al. [25]	NA	NA	NA
Wang et al. [34] (S1)	Tile Type B (32/27), Type C (14/15)	NA	6.95/7.14
Wang et al. [34] (S2)	Tile Type B (17/9), Type C (4/10)	NA	7.15/8.86
Wang et al. [35]	AO Type C1(19/14), Type C2(13/7)	NA	5/5
Zhao et al. [26]	NA	NA	4.29/5.25

The detailed characteristics of pelvic fracture information, including fracture type, comorbidity, and average time from injury to surgery

**Table 4:** Detailed characteristics of outcome information

Author	Outcome assessment
Gu [28]	Operative time, frequency of intraoperative fluoroscopy, blood loss, fracture healing time
Han et al. [32]	Screw position rating, radiation exposure time of screw implantation, guide wire attempts, Matta rating
Hu et al. [27]	Radiation exposure time of screw implantation, frequency of drilling, screw implantation duration, Majeed score, blood loss
Li et al. [29]	Radiation exposure time of screw implantation, operative time, Matta rating, Majeed score, screw position rating
Liu et al. [33]	Operative time, blood loss, fluoroscopy times of fluoroscopy, fracture healing time, Majeed score
Liu et al. [30]	Operative time, intraoperative fluoroscopy time, guide wire attempts, fracture healing time, Majeed score, Matta rating, screw position rating
Long et al. [31]	Frequency of intraoperative fluoroscopy, intraoperative fluoroscopy time, operation time, blood loss, fracture healing time, Matta rating, Majeed score
Wang et al. [25]	Guide wire attempts, operation time, radiation exposure time of screw implantation, screw position rating
Wang et al. [34] (S1)	Radiation exposure time of screw implantation, guide wire attempts, screw position rating, Majeed score and rating
Wang et al. [34] (S2)	Radiation exposure time of screw implantation, guide wire attempts, screw position rating, Majeed score and rating
Wang et al. [35]	Operation time, blood loss, radiation exposure time of screw implantation, screw position rating, Majeed score
Zhao et al. [26]	Radiation exposure time of screw implantation, operation time, screw position rating

### Risk-of-bias assessment

The methodological quality of the involved studies ranged from seven to nine (Table 5). The risk-of-bias summary and risk-of-bias graph for RCTs are presented in Table 6. As a result, the overall quality of the included studies was considered adequate.

**Table 5:** Risk-of-bias assessment for the studies included in the meta-analysis (NOS)

(nRCT) Study = 6	Selection				Comparability	Outcome/exposure			Score
	Item 1	Item 2	Item 3	Item 4	Item 5	Item 6	Item 7	Item 8	
Gu [28]	*		*	*	**	*	*	*	8
Han et al. [32]	*	*	*	*	**	*	*	*	9
Hu et al. [27]	*		*	*	**	*	*	*	8
Li et al. [29]	*		*	*	**	*	*	*	8
Liu et al. [33]	*		*	*	**	*		*	7
Liu et al. [30]	*		*	*	**	*	*	*	8
Long et al. [31]	*		*	*	**	*		*	7
Wang et al. [34]	*	*	*	*	**	*	*	*	9
Wang et al. [35]	*	*	*	*	**	*	*	*	9

The methodological quality of the involved studies ranged from six to eight

**Table 6:** Methodological assessment according to six domains of potential biases (Cochrane Risk-of-Bias Tool)

RCT Study = 2	Random sequence generation	Allocation concealment	Blinding of participants and personnel	Blinding of outcome assessment	Incomplete outcome data	Selective reporting	Other bias
Wang [25]	Low	Low	High	Low	Low	Low	Unclear
Zhao [26]	Low	Low	High	Low	Low	Low	Unclear

The RCTs’ methodological quality and basis were assessed as follows: randomization, allocation concealment, blind method, selective reporting, group similarity at baseline, incomplete outcome data, compliance, the timing of outcome assessments, and intention-to-treat analysis

*RCT* randomized controlled trials

## Outcomes of meta-analysis

### The radiation exposure time of screw implantation

**Fig. 2:**
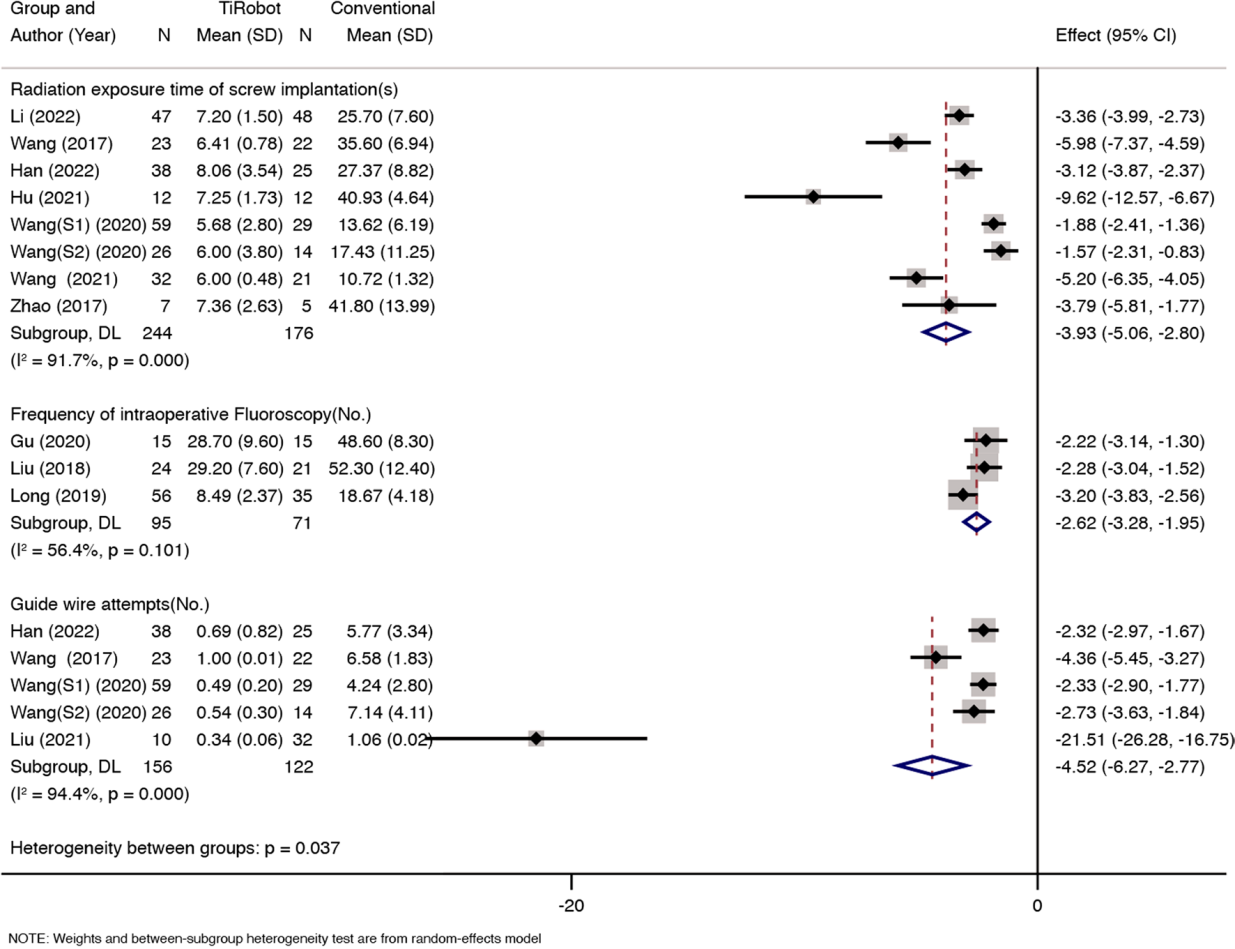
Eight studies reported radiation exposure time of screw implantation; we found statistical heterogeneity between the two groups (*x*^2^ = 83.9, d*f* = 7, *P* = 0.000; *I*^2^ = 91.7%); TiRobot would decrease radiation exposure time of screw implantation [MD = − 3.93, 95% CI (− 5.06, − 2.8), *P* = 0.000]. Three studies reported the frequency of intraoperative fluoroscopy. We found some statistical heterogeneity between the two groups (*x*^2^ = 4.58, d*f* = 2, *P* = 0.101; *I*^2^ = 56.4%). TiRobot would decrease the frequency of intraoperative fluoroscopy [MD = − 2.62, 95% CI (− 3.28, − 1.96), *P* = 0.000]. Five studies reported guide wire attempts. We found some statistical heterogeneity between the two groups (*x*^2^ = 71.62, d*f* = 4, *P* = 0.000; *I*^2^ = 94.4%). TiRobot would decrease guide wire attempts [MD = − 4.52, 95% CI (− 6.27, − 2.77), *P* = 0.000]

Eight studies reported radiation exposure time of screw implantation; we found statistical heterogeneity between the two groups (*x*^2^ = 83.9, d*f* = 7, *P* = 0.000; *I*^2^ = 91.7%; see Fig. 2); thus, a random-effects model was used. The pooled results showed that compared with conventional percutaneous sacroiliac screw fixation, TiRobot would decrease radiation exposure time of screw implantation [MD = − 3.93, 95% CI (− 5.06, − 2.8), *P* = 0.000; Fig. 2].

### Frequency of intraoperative fluoroscopy

Three studies reported the frequency of intraoperative fluoroscopy. We found some statistical heterogeneity between the two groups (*x*^2^ = 4.58, d*f* = 2, *P* = 0.101; *I*^2^ = 56.4%; see Fig. 2), and thus a random-effects model was used. The pooled results showed that compared with conventional percutaneous sacroiliac screw fixation, TiRobot would decrease the frequency of intraoperative fluoroscopy [MD = − 2.62, 95% CI (− 3.28, − 1.96), *P* = 0.000; see Fig. 2].

### Guide wire attempts

Five studies reported guide wire attempts. We found some statistical heterogeneity between the two groups (*x*^2^ = 71.62, d*f* = 4, *P* = 0.000; *I*^2^ = 94.4%; see Fig. 2), and thus a random-effects model was used. The pooled results showed that compared with conventional percutaneous sacroiliac screw fixation, TiRobot would decrease guide wire attempts [MD = − 4.52, 95% CI (− 6.27, − 2.77), *P* = 0.000; see Fig. 2].

### Intraoperative blood loss

**Fig. 3:**
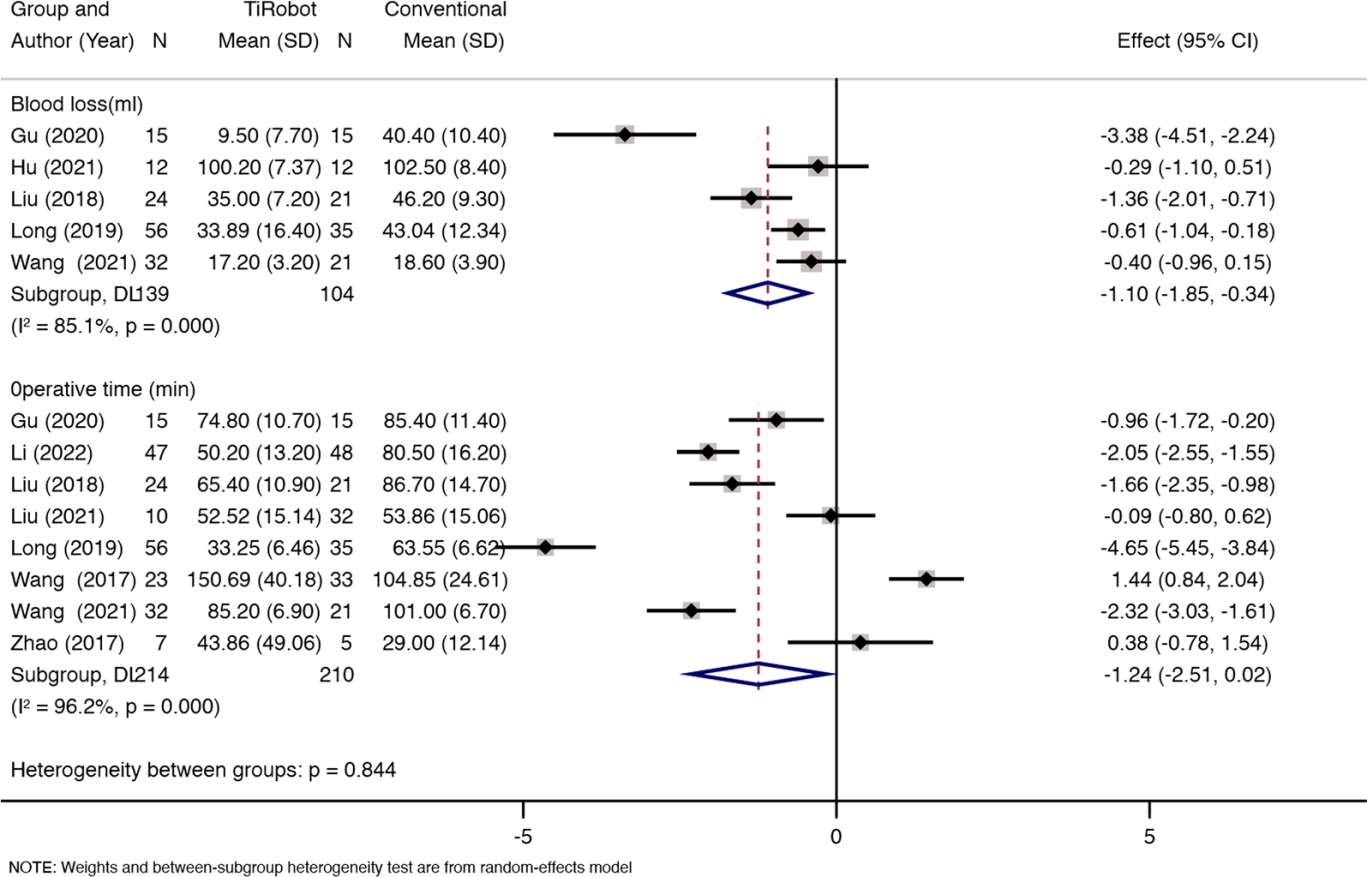
Five studies reported intraoperative blood loss. We found statistical heterogeneity between the two groups (*x*^2^ = 26.76, d*f* = 4, *P* = 0.000; *I*^2^ = 85.1%). TiRobot would decrease intraoperative blood loss [MD = − 1.10, 95% CI (− 1.85, − 0.34), *P* = 0.005]. Eight studies reported operative time. We found statistical heterogeneity between the two groups (*x*^2^ = 184.87, d*f* = 7, *P* = 0.000; *I*^2^ = 96.2%). Both groups experienced a similar operative time (MD = − 1.24, 95% CI (− 2.51, − 0.02), *P* = 0.055]

Five studies reported intraoperative blood loss. We found statistical heterogeneity between the two groups (*x*^2^ = 26.76, d*f* = 4, *P* = 0.000; *I*^2^ = 85.1%; see Fig. 3), and thus a random-effects model was used. The pooled results showed that compared with conventional percutaneous sacroiliac screw fixation, TiRobot would decrease ﻿intraoperative blood loss [MD = − 1.10, 95% CI (− 1.85, − 0.34), *P* = 0.005; see Fig. 3].

### Operative time

Eight studies reported operative time. We found statistical heterogeneity between the two groups (*x*^2^ = 184.87, d*f* = 7, *P* = 0.000; *I*^2^ = 96.2%; see Fig. 3), and thus a random-effects model was used. The pooled results showed that patients in both groups experienced a similar operative time [MD = − 1.24, 95% CI (− 2.51, − 0.02), *P* = 0.055; see Fig. 3].

### Fracture healing time

**Fig. 4:**
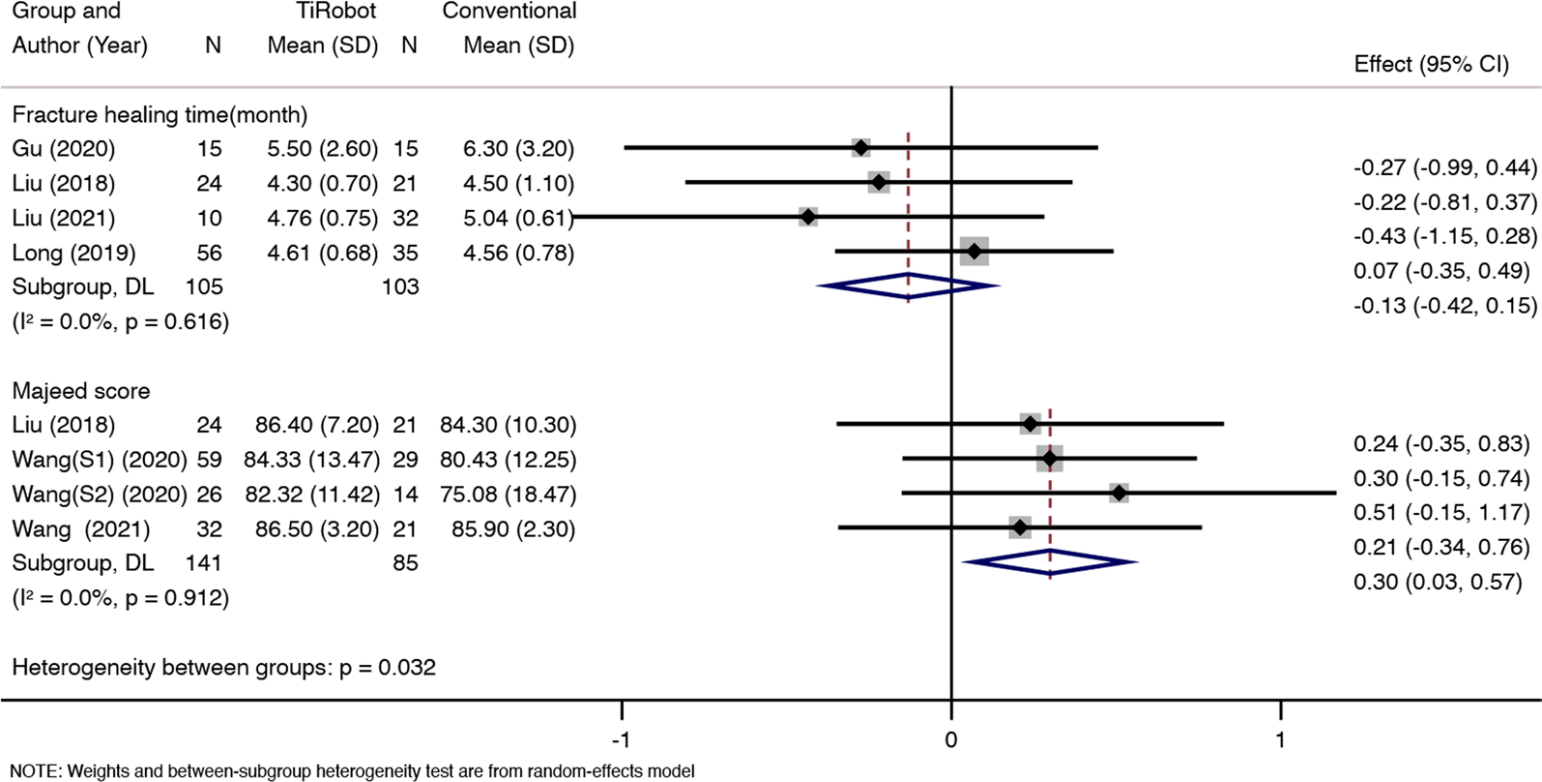
Four studies reported fracture healing time. We did not find statistical heterogeneity between the two groups (*x*^2^ = 1.80, d*f* = 3, *P* = 0.616; *I*^2^ = 0%). Both groups experienced similar fracture healing time [MD = − 0.13, 95% CI (− 0.42, 0.15), *P* = 0.365]. Four studies reported a Majeed score. We did not find statistical heterogeneity between the two groups (*x*^2^ = 0.53, d*f* = 3, *P* = 0.912; *I*^2^ = 0%). TiRobot would improve the Majeed score [MD = 0.3, 95% CI (0.03, 0.57), *P* = 0.031]

Four studies reported fracture healing time. We did not find statistical heterogeneity between the two groups (*x*^2^ = 1.80, d*f* = 3, *P* = 0.616; *I*^2^ = 0%; see Fig. 4), and thus a fixed-effects model was used. The pooled results showed that patients in both groups experienced similar fracture healing time [MD = − 0.13, 95% CI (− 0.42,0.15), *P* = 0.365; see Fig. 4].

### Majeed score

Four studies reported a Majeed score. We did not find statistical heterogeneity between the two groups (*x*^2^ = 0.53, d*f* = 3, *P* = 0.912; *I*^2^ = 0%; see Fig. 4), and thus a fixed-effects model was used. The pooled results showed that compared with conventional percutaneous sacroiliac screw fixation, TiRobot would improve the Majeed score [MD = 0.3, 95% CI (0.03, 0.57), *P* = 0.031; see Fig. 4].

### Overall excellent and good rates of Majeed score

**Fig. 5:**
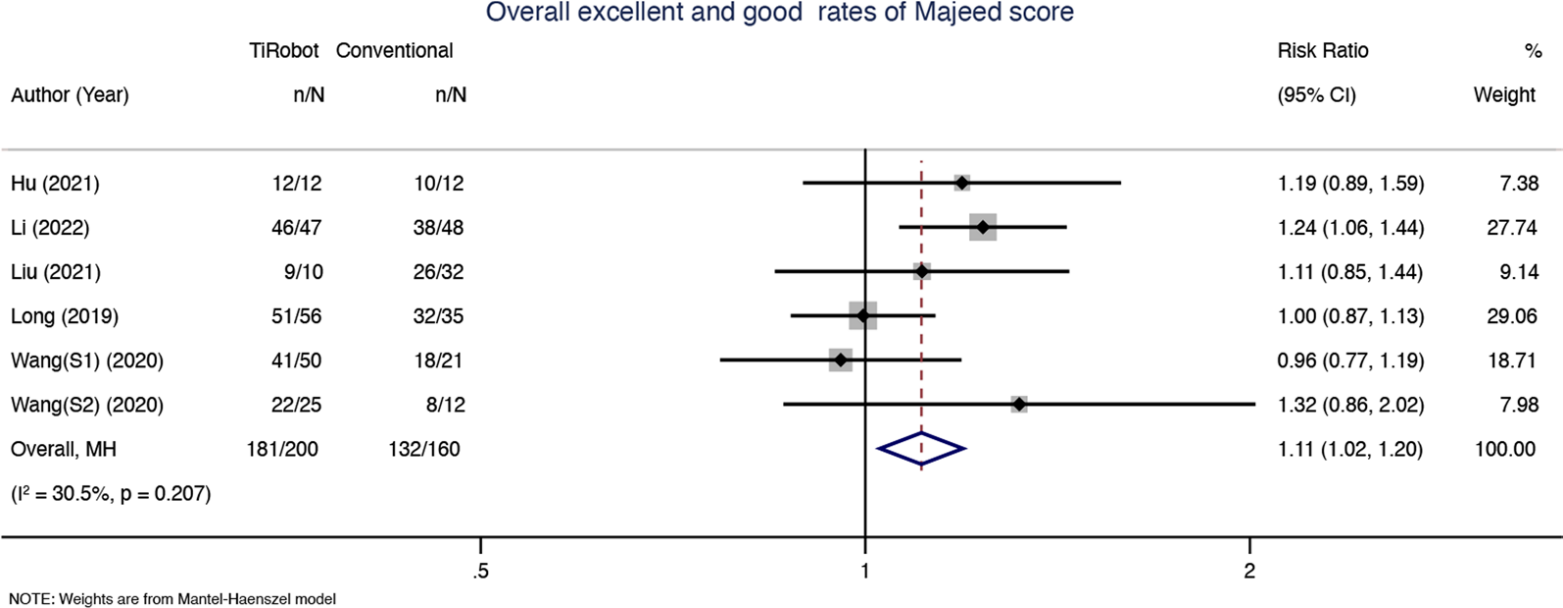
Six studies reported overall excellent and good rates of Majeed score. We did not find high statistical heterogeneity between the two groups (*x*^2^ = 7.19, d*f* = 5, *P* = 0.207; *I*^2^ = 30.5%). TiRobot would have higher overall excellent and good rates of Majeed score [RR = 1.11, 95% CI (1.02, 1.20), *P* = 0.018]

Six studies reported overall excellent and good rates of Majeed score. We did not find high statistical heterogeneity between the two groups (*x*^2^ = 7.19, d*f* = 5, *P* = 0.207; *I*^2^ = 30.5%; see Fig. 5), and thus a fixed-effects model was used. The pooled results showed that compared with conventional percutaneous sacroiliac screw fixation, TiRobot has higher overall excellent and good rates of Majeed score [RR = 1.11, 95% CI (1.02, 1.20), *P* = 0.018; see Fig. 5].

### Overall excellent and good rate of screw accuracy

**Fig. 6:**
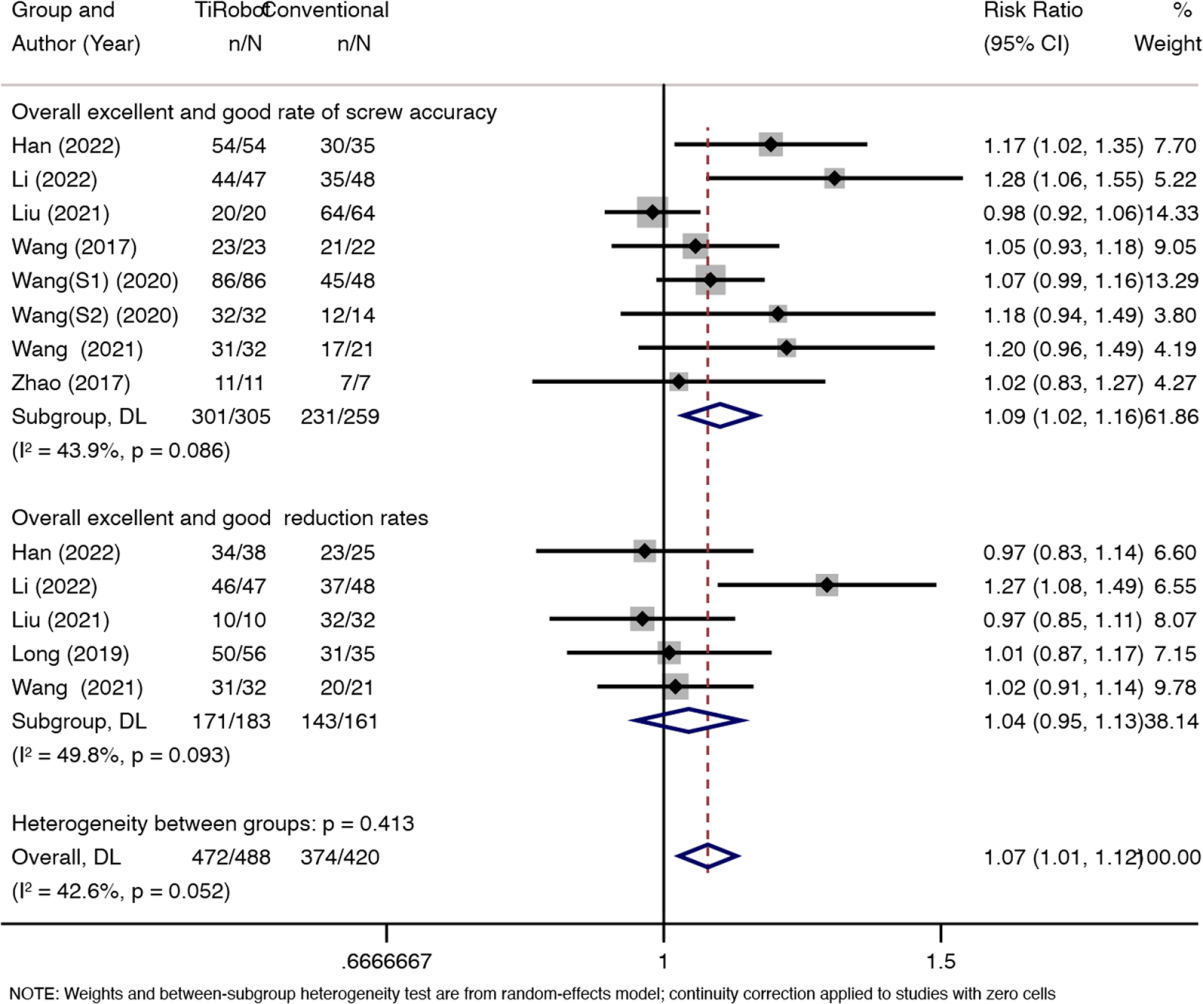
Eight studies reported an overall excellent and good rate of screw accuracy. We found some statistical heterogeneity between the two groups (*x*^2^ = 12.49, d*f* = 7, *P* = 0.086; *I*^2^ = 43.9%). TiRobot would improve screw accuracy [RR = 1.09, 95% CI (1.02, 1.16), *P* = 0.011]. Five studies reported an overall excellent and good rate of reduction. We found some statistical heterogeneity between the two groups (*x*^2^ = 7.97, d*f* = 4, *P* = 0.093; *I*^2^ = 49.8%). Both groups experienced a similar excellent and good rate of reduction accuracy [RR = 1.04, 95% CI (0.95, 1.13), *P* = 0.426]

Eight studies reported an overall excellent and good rate of screw accuracy. We found some statistical heterogeneity between the two groups (*x*^2^ = 12.49, d*f* = 7, *P* = 0.086; *I*^2^ = 43.9%; see Fig. 6), and thus a random-effects model was used. The pooled results showed that compared with conventional percutaneous sacroiliac screw fixation, TiRobot would improve screw accuracy [RR = 1.09, 95% CI (1.02, 1.16), *P* = 0.011; see Fig. 6].

### Overall excellent and good rate of reduction

Five studies reported an overall excellent and good rate of reduction. We found some statistical heterogeneity between the two groups (*x*^2^ = 7.97, d*f* = 4, *P* = 0.093; *I*^2^ = 49.8%; see Fig. 6), and thus a random-effects model was used. The pooled results showed that patients in both groups experienced a similar excellent and good rate of reduction accuracy [RR = 1.04, 95% CI (0.95, 1.13), *P* = 0.426; see Fig. 6].

## Discussion

The TiRobot system comprises surgical planning and robot control software, a robot, spatial calibration components, an optical tracking system, a central control station, and matching tools. The operation process of the robot consists of four steps: ① Device connection and skin disinfection: The equipment was placed and connected; then, the skin on the pelvis was sterilized. The surgeon put a tracer on the opposite side of the anterior superior iliac spine. The surgeon placed a sterile C-type arm sleeve on the mechanical arm and connected a positioning device. ② Fluoroscopy: The C-arm X-ray machine fluoroscopy is used for pelvis inlet and outlet view, ensuring all ten markers on the positioning device are visible. ③ Surgical planning and mechanical arm movement: Surgical planning was carried out based on the inlet and outlet view to design the percutaneous sacroiliac screw point, angle, and length. After the surgical planning, the manipulator's arm moves the pilot sleeve according to the planned path, which is the percutaneous sacroiliac screw entry point and angle. ④ Screw-setting: The surgeon inserted the Kirschner wire along the pilot sleeve and then inserted the sacroiliac screw along the Kirschner wire. The screw position was confirmed by the C-arm X-ray machine.

There is only one meta-analysis comparing TiRobot‑assisted versus conventional fluoroscopy-assisted percutaneous sacroiliac screw fixation for pelvic ring injuries. However, all included studies in the previous meta-analysis were observational studies, the number of the included studies was too small (only four articles), and all included studies were English articles. Furthermore, the previous meta-analysis mixed radiation exposure time (seconds) and frequency of intraoperative fluoroscopy (No.) together that may have high heterogeneity. The strengths of our meta-analysis included: First, we included not only English studies but also Chinese studies. Considering that the TiRobot is only used in China, Chinese-language studies were also very important for the meta-analysis. Second, our meta-analysis included more studies (628 patients in 11 studies). Third, our results added pooled analysis of screw accuracy, reduction accuracy, and overall excellent and good rates of Majeed score, which could be a supplement for the previous meta-analysis. Fourth, we analyzed radiation exposure time (seconds) and frequency of intraoperative fluoroscopy (No.) separately, which would provide a more exact conclusion.

Like the previous meta-analyses, our meta-analysis also found that the TiRobot will result in fewer guide wire attempts, less ﻿intraoperative blood loss, and better screw accuracy. There were no significant differences in terms of fracture healing time. However, our study came to a different conclusion in terms of Majeed score and operative time. We found that the TiRobot will improve the Majeed score, and we did not see a significant difference in operative time. For some newly added outcomes, we found TiRobot will result in less radiation exposure time of screw implantation, less frequency of intraoperative fluoroscopy, and higher overall excellent and good rates of Majeed score and screw accuracy. We did not find a significant difference in terms of reduction accuracy.

Our meta-analysis showed that TiRobot resulted in less radiation exposure time of screw implantation, less frequency of intraoperative fluoroscopy, and guide wire attempts. The real‐time optical tracking technology of TiRobot makes the repetition of X-rays unnecessary during the operation, decreasing the fluoroscopy frequency and the fluoroscopy time and improving the guide wire insertions. However, we must admit that total radiation exposure time includes not only the evaluated “radiation exposure time of screw implantation(s)” but also the total radiation exposure time, which is needed to prepare the robot procedure before the screw insertion. Before the screw implantation, the surgeon needs to use the C-arm X-ray machine fluoroscopy for pelvis inlet and outlet view, ensuring all ten markers on the positioning device are visible. This step is an extra radiation exposure step compared to conventional groups. We think although the Robot-assisted system needs fluoroscopy for the screw position planning, the overall radiation exposure of both the patients and the surgical team was still lower, providing a more significant safety level. The robotic arm can significantly reduce the deviation caused by the instability of human manual operation, improve the accuracy and safety of the process, and reduce the secondary damage caused by repeated needle insertion in traditional surgery.

The evidence based on this current study confirms that there may be no difference in operative time between the two groups, which is inconsistent with the previous meta-analysis. Theoretically, less radiation exposure time of screw implantation, less frequency of intraoperative fluoroscopy, and guide wire attempts will shorten the operation time and improve the operation efficiency. However, the operation time of the robot system includes the noninvasive time of the robot system and the invasive time of the actual operation. Steps such as collecting fluoroscopic images, positioning the cursor, and planning are extra steps compared to a conventional group and therefore take time. The real invasive operation time is only about 20 min. We also cannot ignore the factors of proficiency, the surgeon's experience, and the operation team's tacit understanding. It is believed that by improving the proficiency in the operation process of the surgical robot-assisted navigation system, surgeons can further shorten noninvasive operation time and the overall operation time. This discrepancy may be because we included some earlier studies in which surgeons lacked expertise in utilizing the robot-assisted system in the early period. With the experience, proficiency, and degree of tacit understanding of cooperation gradually increasing, the surgeons can significantly shorten the duration of operations.

Intraoperative blood loss was notably lower in the TiRobot group, potentially due to the reduced incision length [31, 33], reduced guide wire attempts, and better precision of screw placement. Although there was a statistically significant difference in ﻿intraoperative blood loss with the robot group relative to the conventional group, it is unknown whether this statistical difference is clinically meaningful. As well as the length of incision, this may suggest less damage to patients, and the use of TiRobot may be more conducive to postoperative recovery.

There is no significant difference in fracture healing time between the two groups. Despite TiRobot offering better screw accuracy and less radiation exposure; it does not change the degree of comminution, reduction accuracy, and the pelvis’s blood supply, which plays a crucial role in healing duration [36]

Accurate screw path planning and position are crucial to achieving enhanced outcomes in robot-assisted fixation [31]. Screw positioning, evaluated by Florian Gras classification, was notably improved in the TiRobot group in our meta-analysis. Reduction is a crucial step in the surgical treatment of bone fractures to achieve anatomical alignment and facilitate healing [37, 38]. The Matta score evaluates the reduction quality of pelvic fractures [38]. Our meta-analysis found no significant difference between overall excellent and good rate of reduction accuracy according to Matta criteria. Although TiRobot provides real‐time optical tracking technology, it does not change the reduction manner.

Postoperative outcomes measured using Majeed scores have shown more favorable results in the TiRobot group, inconsistent with the previous meta-analysis. The previous meta-analysis didn't find a significant difference in Majeed scores. It seems better accuracy of screw placement positively affects postoperative outcomes [39]. We think the reason for this discrepancy may be the increased number of the included studies in our meta-analysis that can give a significant difference conclusion. However, this result may have some limitations, because the Majeed scores in our included studies were assessed at different follow-up times. In Liu’s study [33], the mean follow‐up duration was 5.4 months (4–12 months). In Wang’s study [34], the mean follow-up time was 11.23 months (5–20 months). In Wang’s study [35], the mean follow-up time was 13.2 months (12–15 months).

We must admit that orthopedic robot systems are expensive and require complete supporting facilities. It is difficult for grassroots hospitals to implement them. Current initiatives should focus not only on improving operative outcomes but also on lowering costs. Potential economic savings gained by using robots in pelvic fracture surgery can be estimated by considering the decreased operative time, patient length of stay, fluoroscopic exposure, and surgical revision rates that robots may increasingly provide in the future. Reduced revision pelvic fracture surgery rates may ultimately be the most effective means of cost savings in this patient population due to the increased direct costs of additional surgery and the indirect cost of prolonged patient disability.

We should consider several limitations before interpreting these findings. First, some studies with a small sample size may lead to unreliable results during meta-analysis. Second, we only included two randomized controlled trials; the other nine studies were observational studies, which may have reduced the quality of the evidence for this meta-analysis. Although we have included all related studies thus far and tried to collect more data to make this meta-analysis, more prospective randomized trials are needed to confirm the results and conclusions. Third, none of the studies evaluated the cost-effectiveness of TiRobot. Cost is the main barrier to robot-assisted surgery, which includes the devices and the associated learning curve and surgeon training. They may be more feasible in large hospitals with a large number of patients [27]. We hope that future studies focus on the overall cost of adopting TiRobot and other systems. Fourth, we didn't compare complications between the two groups because too many factors can affect the occurrence of complications, making the complication comparison high heterogeneity.

## Conclusion

TiRobot-assisted fixation has less intraoperative fluoroscopy and intraoperative blood loss, superior screw accuracy, and postoperative outcomes compared with conventional fixation. TiRobot also has no significant effect on operative time, fracture healing time, and reduction accuracy. Given the relevant possible biases in our meta-analysis, we required more adequately powered and better-designed RCT studies with long-term follow-up to reach a firmer conclusion.

## Data Availability

The datasets generated during and/or analyzed during the current study are available from the corresponding author upon reasonable request.

## Abbreviations

CIs: Confidence intervals
RCTs: Randomized controlled trials
RCS: Retrospective cohort study
PCS: Prospective cohort study
RR: Risk ratio
OR: Odds ratio
VMD: Weighted mean difference
BMI: Body mass index
EMBASE: Excerpta Medica dataBASE
CENTRAL: Cochrane Central Register of Controlled Trials
CNKI: China National Knowledge Infrastructure

## References

[1] Burkhardt M, Kristen A, Culemann U, Koehler D, Histing T, Holstein JH (2014). Pelvic fracture in multiple trauma: Are we still up-to-date with massive fluid resuscitation?. Injury.

[2] Hermans E, Edwards MJR, Goslings JC, Biert J (2018). Open pelvic fracture: The killing fracture?. J Orthop Surg Res.

[3] Routt ML, Nork SE, Mills WJ (2000). Percutaneous fixation of pelvic ring disruptions. Clin Orthop Relat Res..

[4] Leone E, Garipoli A, Ripani U, Lanzetti RM, Spoliti M, Creta D (2022). Imaging review of pelvic ring fractures and its complications in high-energy trauma. Diagnostics (Basel).

[5] Patel S, Aggarwal S, Jindal K, Kumar V, Sharma S (2022). Outcomes and complications of the INFIX technique for unstable pelvic ring injuries with high-velocity trauma: a systematic review and meta-analysis. Arch Orthop Trauma Surg.

[6] Kazley JM, Potenza MA, Marthy AG, Arain AR, O’Connor CM, Czajka CM (2020). Team approach: evaluation and management of pelvic ring injuries. JBJS Rev.

[7] Zheng J, Feng X, Xiang J, Liu F, Leung FKL, Chen B (2021). S2-alar-iliac screw and S1 pedicle screw fixation for the treatment of non-osteoporotic sacral fractures: a finite element study. J Orthop Surg Res.

[8] Templeman D, Schmidt A, Freeze J, Weisman I (1996). Proximity of iliosacral screws to neurovascular structures after internal fixation. Clin Orthop Relat Res.

[9] Hinsche AF, Giannoudis PV, Smith RM (2002). Fluoroscopy-based multiplanar image guidance for insertion of sacroiliac screws. Clin Orthop Relat Res.

[10] Mathew G, Hanson BP (2009). Global burden of trauma: Need for effective fracture therapies. Indian J Orthop.

[11] Yin Y, Zhang R, Li S, Chen W, Zhang Y, Hou Z (2019). Computational analysis on the feasibility of transverse iliosacral screw fixation for different sacral segments. Int Orthop.

[12] Miller AN, Routt MLC (2012). Variations in sacral morphology and implications for iliosacral screw fixation. J Am Acad Orthop Surg.

[13] Eastman JG, Chip Routt ML (2015). Correlating preoperative imaging with intraoperative fluoroscopy in iliosacral screw placement. J Orthop Traumatol.

[14] Sun C, Yang K, Li H, Cai X (2018). Application of robot system in knee arthroplasty. Chin Med J.

[15] Sun C, Yang K, Li H, Cai X (2018). Application of robot system in total hip arthroplsty. Chin Med J.

[16] Lee NJ, Buchanan IA, Boddapati V, Mathew J, Marciano G, Park PJ (2021). Do robot-related complications influence 1 year reoperations and other clinical outcomes after robot-assisted lumbar arthrodesis? A multicenter assessment of 320 patients. J Orthop Surg Res.

[17] Wang B, Cao J, Chang J, Yin G, Cai W, Li Q (2021). Effectiveness of Tirobot-assisted vertebroplasty in treating thoracolumbar osteoporotic compression fracture. J Orthop Surg Res.

[18] Christ AB, Hansen DG, Healey JH, Fabbri N (2021). Computer-assisted surgical navigation for primary and metastatic bone malignancy of the pelvis: current evidence and future directions. Hss J.

[19] Türkay S, Letheren K, Crawford R, Roberts J, Jaiprakash AT (2021). The effects of gender, age, and videogame experience on performance and experiences with a surgical robotic arm: an exploratory study with general public. J Robot Surg.

[20] Peng Y, Zhang W, Zhang G, Wang X, Zhang S, Ma X (2019). Using the Starr Frame and Da Vinci surgery system for pelvic fracture and sacral nerve injury. J Orthop Surg Res.

[21] Shamseer L, Moher D, Clarke M, Ghersi D, Liberati A, Petticrew M (2015). Preferred reporting items for systematic review and meta-analysis protocols (PRISMA-P) 2015: elaboration and explanation. BMJ.

[22] Stang A (2010). Critical evaluation of the Newcastle-Ottawa scale for the assessment of the quality of nonrandomized studies in meta-analyses. Eur J Epidemiol.

[23] Higgins JPT, Altman DG, Gøtzsche PC, Jüni P, Moher D, Oxman AD (2011). The Cochrane collaboration’s tool for assessing risk of bias in randomised trials. BMJ.

[24] Hozo SP, Djulbegovic B, Hozo I (2005). Estimating the mean and variance from the median, range, and the size of a sample. BMC Med Res Methodol.

[25] Wang J-Q, Wang Y, Feng Y, Han W, Su Y-G, Liu W-Y (2017). Percutaneous sacroiliac screw placement: a prospective randomized comparison of robot-assisted navigation procedures with a conventional technique. Chin Med J.

[26] Chunpeng Z, Wang Junqiang Su, Yonggang WH, Li Z, Manyi W (2017). Robot-assisted percutaneous screw fixation for pelvic and acetabular fractures. J Peking Univ (Health Sci).

[27] Hu Haonan. Related research on orthopedic robot-assisted sacroiliac joint screw placement in the treatment of sacral fractures [Internet] [硕士]. Inner Mongolia Medical university; 2021. Available from: https://kns.cnki.net/KCMS/detail/detail.aspx?dbcode=CMFD&dbname=CMFDTEMP&filename=1021814042.nh&v=

[28] Shanling Gu, Yan Xu, Gang Y, Yan L (2020). Efficacy of orthopedic surgery robot-assisted percutaneous screw internal fixation in the treatment of elderly pelvic and acetabular fractures and its impact on quality of life. Chin J Gerontol.

[29] Li C, Wu Z, Zeng Y, He Y, Xiaopeng S, Du X (2022). Analysis of the advantages and disadvantages of robotic-assisted and traditional fluoroscopic percutaneous sacroiliac screw placement in orthopedic surgery. J Clin Rehabilit Tissue Eng Res.

[30] Liu C, Huang Z, Tang J, Zhou D, He J, Hu J (2021). Comparison of orthopedic surgical robot and “O”-arm X-ray navigation assisted percutaneous internal fixation of pelvic fractures. Chin J Orthop.

[31] Long T, Li K, Gao J, Liu T, Mu J, Wang X (2019). Comparative study of percutaneous sacroiliac screw with or without TiRobot assistance for treating pelvic posterior ring fractures. Orthop Surg.

[32] Han W, Zhang T, Su Y, Zhao C, Zhou L, Wu X (2022). Percutaneous robot-assisted *versus* Freehand S_2_ iliosacral screw fixation in unstable posterior pelvic ring fracture. Orthop Surg.

[33] Liu HS, Duan SJ, Liu SD, Jia FS, Zhu LM, Liu MC (2018). Robot-assisted percutaneous screw placement combined with pelvic internal fixator for minimally invasive treatment of unstable pelvic ring fractures. Int J Med Robot.

[34] Wang J, Zhang T, Han W, Hua K, Wu X (2020). Robot-assisted S2 screw fixation for posterior pelvic ring injury. Injury.

[35] Tianlong W, Zifei Z, Junfeng L, Longpo Z (2021). Curative effect of TiRobot robot combined with O-arm X-ray machine in minimally invasive treatment of C-type pelvic fractures. Chin J Trauma.

[36] Matityahu A, Marmor M, Elson JK, Lieber C, Rogalski G, Lin C (2013). Acute complications of patients with pelvic fractures after pelvic angiographic embolization. Clin Orthop Relat Res.

[37] Zhao C, Wang Y, Wu X, Zhu G, Shi S (2022). Design and evaluation of an intelligent reduction robot system for the minimally invasive reduction in pelvic fractures. J Orthop Surg Res.

[38] Lai C-Y, Lai P-J, Tseng I-C, Su C-Y, Hsu Y-H, Chou Y-C (2022). Postoperative reduction quality may be the most important factor that causes worse functional outcomes in open and closed pelvic fractures. World J Surg.

[39] Banierink H, Meesters AML, Ten Duis K, Doornberg JN, El Moumni M, Heineman E (2021). Does 3D-assisted operative treatment of pelvic ring injuries improve patient outcome?-A systematic review of the literature. J Pers Med.

